# LitCovid-AGAC: cellular and molecular level annotation data set based on COVID-19

**DOI:** 10.5808/gi.21013

**Published:** 2021-09-30

**Authors:** Sizhuo Ouyang, Yuxing Wang, Kaiyin Zhou, Jingbo Xia

**Affiliations:** Hubei Key Lab of Agricultural Bioinformatics, College of Informatics, Huazhong Agricultural University, 430070 Wuhan, China

**Keywords:** AGAC, annotation, corpus, knowledge discovery, LitCovid

## Abstract

Currently, coronavirus disease 2019 (COVID-19) literature has been increasing dramatically, and the increased text amount make it possible to perform large scale text mining and knowledge discovery. Therefore, curation of these texts becomes a crucial issue for Bio-medical Natural Language Processing (BioNLP) community, so as to retrieve the important information about the mechanism of COVID-19. PubAnnotation is an aligned annotation system which provides an efficient platform for biological curators to upload their annotations or merge other external annotations. Inspired by the integration among multiple useful COVID-19 annotations, we merged three annotations resources to LitCovid data set, and constructed a cross-annotated corpus, LitCovid-AGAC. This corpus consists of 12 labels including Mutation, Species, Gene, Disease from PubTator, GO, CHEBI from OGER, Var, MPA, CPA, NegReg, PosReg, Reg from AGAC, upon 50,018 COVID-19 abstracts in LitCovid. Contain sufficient abundant information being possible to unveil the hidden knowledge in the pathological mechanism of COVID-19.

## Introduction

Coronavirus disease 2019 (COVID-19) is an abbreviation for corona virus disease, which caused a pandemic in 2019. People infected with COVID-19 suffers from severe high fever, dyspnea, lung disease and with 0.3%‒1.5% chance of death. Due to the severe condition COVID-19 caused, the research upon the disease has been increasing dramatically. As of January 2021, there are over 90,000 related literature published, and make it a huge repository for knowledge discovery. Such a large growth rate makes it difficult for relevant researchers to understand the massive information in time.

Understanding the mechanism of COVID-19 is of importance for containing the virus. Like severe acute respiratory syndrom virus, it enters cells by binding angiotensin-converting enzyme 2 (ACE2) protein on the surface of human cells with S protein. S protein is located in the outermost layer of COVID-19, and exists in the form of trimer. Each monomer contains a receptor binding domain composed of amino acids where S protein binds to ACE2 and infects human cells.

Compared with the whole vision of the COVID-19 mechanism, the above commonsense knowledge is far from sufficiency. For unveiling the mechanism hidden in the huge text data, application of text mining has drawn a good amount of attentions recently. So far, nearly 200 researches have been published in PubMed, which worked on COVID-19 literature mining. For propelling the COVID-19‒oriented text mining researches, NCBI developed a huge public available COVID-19 corpus, LitCovid [1,2], and make it a gold database for knowledge mining.

Fortunately, the Bio-medical Natural Language Processing (BioNLP) community has long focused on fundamental tools development, including bio-medical entity recognition, entity concept normalization, relation extraction, and so forth. For PubMed abstracts and PMC full texts, PubTator [3] efficiently tags and normalizes six types of biological entities, i.e., gene, disease, chemical, mutation, species and cell line.

For example, PubTator is a search database that highlights some keywords in the search results, it's based on the results of PubMed. Pubtator supports six tag types, which are gene, disease, chemical, mutation, species and cell line. The above six kinds of tags are already very useful for unveil hidden mechanism of COVID-19. LitCovid is a reliable corpus which is a collection of texts related to COVID-19. Therefore, when PubTator annotates the LitCovid corpus, the six biological entities in the text will be assigned a corresponding tag. Moreover, the OntoGene’s Bio-medical Entity Recognizer (OGER) [4,5] is an important Tagger, which will annotate the following seven bio-medical entities, Disease, Chemical, Sequence, Gene/Protein, Biological_process, Organism and Cell, and these were annotated by using Bio Term Hub (BTH) terminologies. BTH supports the rapid construction of term resources from famous life science databases in a simple standardized format for text mining, and it can label specific concept types such as protein, gene, disease and cell line. However, we use OGER only to add gene ontology (GO) and chemical annotations to our data set.

Considering the need for logical mining, AGAC is good at discovering Regulation relations. Therefore, it is easy to reveal Pathway-like logic. In this research, we release LitCovid-AGAC database. It provides multiple annotations by PubTator, OGER and AGAC.

## Methods

### AGAC as a corpus for key annotations labeling

The purpose of designing AGAC [6] corpus is to better find the logical lines in the sentence, and designed six tags for this, namely Var, MPA, CPA, PosReg, NegReg, Reg. It took 20 months for 4 annotators to manually annotate and check. AGAC is illuminative to be applied in drug-related knowledge discovery. For example, AGAC was successfully applied in LOF/GOF classification by using tensor decomposition algorithm [7]. As well, it has been adopted as the training data in a competition in the BioNLP open shared task 2019 [8], and applied to extract relevant literature for Alzheimer’s disease for the support of gene disease association prediction [9].

### AGAC tagger

An AGAC tagger based on the deep neural network was introduced as a baseline method in AGAC track in BioNLP OST 2019. The baseline fully used sophisticated BERT structure and reached sufficient high quality for sequence labeling [7], the F-1 value of which is about 0.5. Such high-quality annotation results indicate that applying AGAC corpus to annotate the text helps to find the convincing logical relationships between biological entities.

### PubAnnotation platform for multiple annotations alignment

PubAnnotation [10] is a platform for biologist curator to assemble annotations or annotate their own labels upon interested texts. Till now, there are 45 released projects in PubAnnotation with AGAC included. Co-tagging is possible to carry on automatically via PubAnnotation, as various bio concept taggers, e.g., OGER and PubTator, have already been involved in the system. Co-tagging helps to integrate different annotations and to serve sophisticated knowledge representation. As can be seen from the following example, three resources mentioned above provided different kinds of annotation on a same sentence, which form a complete logical line shown in the figure.

**Fig. 1.**
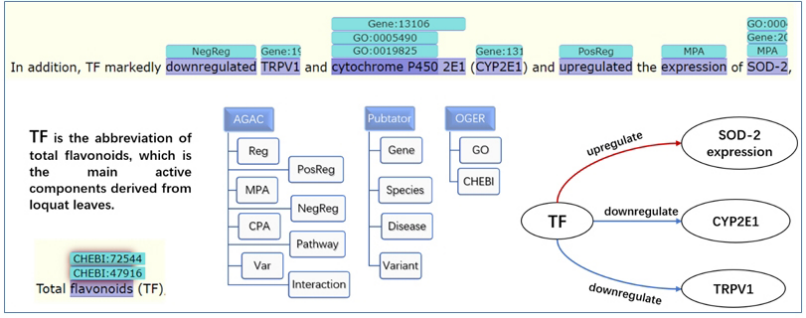
The knowledge representation based on the LitCovid-AGAC corpus.

As shown in Fig. 1, TF is the abbreviation of total flavonoid, which has been labeled in the previous article. By combining AGAC labels with other important annotations, we can clearly see the logical lines shown at the bottom right of the figure. The data we uploaded can be downloaded in PubAnnotation in JSON format. The annotation set we released combines the annotation of PubTator, OGER and AGAC, which can be used to mine the logical lines of biological process changes in COVID-19.

It can be seen that different corpora have different annotation focuses. Other corpora mainly label biological concepts and match them to standard data sets. However, AGAC not only focuses on biological concepts, but also focuses on logical lines in sentences. The same biological concept may be given different labels in different contexts, or even will not be labeled. In this way, we can find that some chemicals up regulate or down regulate gene expression in COVID-19.

### Automatic annotation pipeline

By integrating the method mentioned above, we performed an automatic annotation pipeline to obtain the LitCovid-AGAC dataset.

Step 1. Data collection: Collect literature data set from LitCovid [1,2].

Step 2. AGAC annotation: Obtain the AGAC annotations by applying AGAC tagger on literature set.

Step 3. Regulation annotation: Create a regulation dictionary on PubDictionary [10] and automatically annotate the regulation words.

Step 4. PubTator and OGER annotation: Import the annotations from PubTator and OGER by using PubAnnotation.

## Results

### Statistics of LitCovid-AGAC dataset

LitCovid-AGAC contains 50,018 abstracts from PubMed, and the annotations are from three sources, AGAC, PubTator and OGER. LitCovid-AGAC aims on the regulations of biological process described in COVID-19 literature. Therefore, we applied all the AGAC labels which contains 5 biological concept labels and 3 regulation labels. To enrich the relative annotation, Mutation, Species, Gene, Disease from PubTator and GO, Chemical Entities of Biological Interest (CHEBI) [11] from OGER are included in LitCovid-AGAC dataset. CHEBI includes natural products and synthetic products used to intervene in biological processes, but generally does not include macromolecules encoded by genes. According to the statistics data, the most frequent label is “Disease,” which appears 285,135 times, and the least frequent label is "Mutation", which only appears 435 times.

It can be clearly seen that the annotation results of OGER and PubTator are more abundant, on the contrary, the number of AGAC annotations is not in the same order of magnitude as the number of their annotations. It is due to the annotation rules in AGAC that the sentence without the description of regulation is not annotated, so AGAC annotations are less than the annotations from other sources. The more detailed statistics is shown in Table 1.

### Knowledge discovery pattern and research paradigm in LitCovid-AGAC dataset

#### Logical line examples from single sentence in cellular and molecular level

**Fig. 2.**
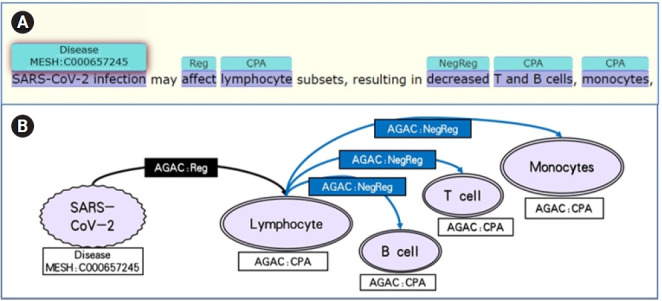
(A, B) A cellular level annotation example of LitCovid-AGAC data set.

Enriched by PubTator and OGER, the data set contained more complete annotations. For instance, in Fig. 2A, the "Disease" annotation provided by PubTator acts as the cause among the other annotations from AGAC in this sentence, where the “Cell Physiological Activity,” lymphocyte, was firstly regulated by the severe acute respiratory syndrom coronavirus 2 (SARS-CoV-2) infection and other two “Cell Physiological Activity,” T and B cells and monocytes, were down-regulated subsequently. From the annotations in this sentence, the effect of the SARS-CoV-2 infection in cell level was clearly showed with the sequential order, which could be transformed to a path in a knowledge graph.

**Fig. 3.**
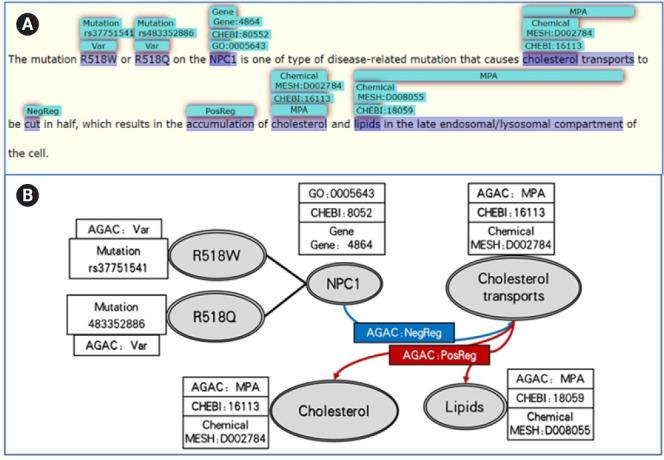
(A, B) A molecular level annotation example of LitCovid-AGAC data set.

Besides, the annotations also unveil the molecule-level biological processes. In Fig. 3, R518W/Q mutations in gene NPC1 inhibited the cholesterol transports and thus resulted the accumulation of cholesterol and lipids, which are all “Molecular Physiological Activity.” In this sentence, AGAC annotations provided the variation, regulation and molecular level processes, while PubTator and OGER provided the gene, variation, chemical and GO [12] annotations with their unique ID which supplemented the information recognition and also provided the normalization on some of the AGAC annotations. GO has three categories, which are biological process, molecular function and cellular component, these terms used to represent all entities and their relationships.

With the annotations in LitCovid-AGAC data set, the genes, diseases, variations and the biological processes in cellular-level and molecular-level are connected by the regulations 4 labels in the same sentence. Combining with the semantics information, the sequential order of the regulation events helps to convert them into a directional path which regards regulation label as the edges and the other labels as the nodes. For example, the path in Fig. 2B is a neutral regulation edge from SARS-CoV-2 infection to lymphocytes then three negative regulations to T and B cells and monocytes. The same knowledge pattern is shown in Fig. 3B. The numerous knowledge paths in this data set are able to construct a network with plenty of biological information contained in the COVID-19 literature, which should contribute to the pathological mechanism analysis of COVID-19 and the evolution of this virus.

#### Combined logical lines from multiple sentences

**Fig. 4.**
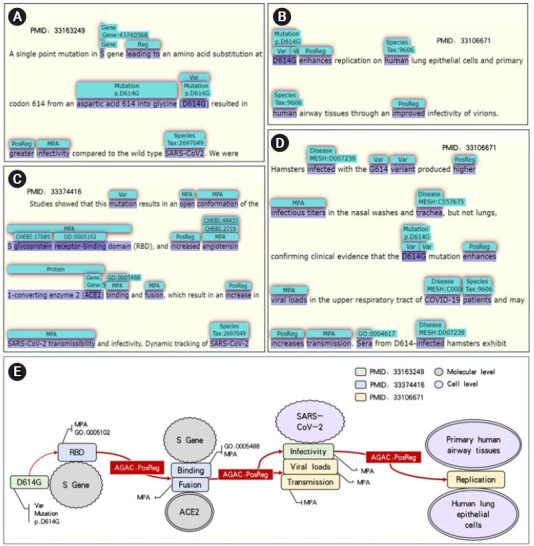
(A‒E) A light logical network inferred from LitCovid-AGAC data set.

Combining the annotations in different articles can get a complete logical line. The D614G mutation of spike gene (S gene) in Fig. 4A will lead to greater infectivity of SARS-CoV-2 virus. The information in Fig. 4C and 4D shows that this mutation leads to the open conformation change of S-glycoprotein receptor-binding domain. It also enhances the viral loads of upper respiratory tract and the binding and fusion of ACE2 in patients with COVID-19, which increased the spread of SARS-CoV-2 virus, resulting in the enhancement of the replication of human lung epithelial cells and primary human airway tissues as shown in Fig. 4B.

Combined with the contents of four pictures, we drew the Fig. 4E, which shows the logical lines that are contained in the four examples above. Fig. 4A only shows that D614G mutation will lead to higher infectivity of SARS-CoV-2 virus, but the addition of D614G mutation in Fig. 4C and 4D will lead to the enhancement of ACE2 binding and fusion, which makes SARS-CoV-2 virus produce more virus transmission and viral loads. Therefore, the logical relationship from S gene to ACE2 to SARS-CoV-2 was formed. As the virus infectivity increasing, a series of immune reactions will appear in the patients’ body infected with SARS-CoV-2. This information is supplemented in Fig. 4B.

This example reflects not only the information at the molecular level, but also the information at the cellular level, which proves the feasibility of finding and forming a logical line from different texts. Therefore, an idea can be put forward that we can extract the key knowledge from the massive information and form a large logical network when the number of texts is enough. As a result, more hidden information can be discovered and new knowledge can be inferred.

## Discussion

As indicated in this research, though single annotation is limited for comprehensive bio-medical knowledge discovery upon the huge literature repository for COVID-19, combination of relevant annotations from different resources makes it possible to bring a rich annotation data set which lead to knowledge with complete semantics.

Furthermore, the suggested knowledge pattern by using LitCovid-AGAC is capable of offering a huge amount of structured logic knowledge, and unveiling the pathological mechanism of COVID-19 in cellular or molecular level.

**Fig. 5.**
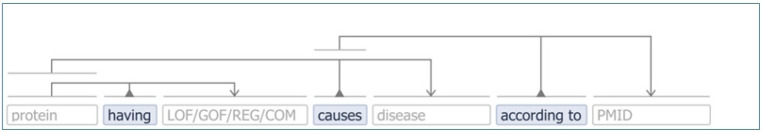
Visualization semantic structure template.

In addition, it as well makes sense to further curate the obtained results in LitCovid-AGAC, e.g., concept normalization, co-reference, and relation extraction. Meanwhile, it is instructive to visualize the knowledge entry in a syntactic way. The VSM box [13] in Fig. 5 presents a typical knowledge template which carries a type of semantic structure of the information in LitCovid-AGAC. The LOF/GOF/REG/COM can be inferred from the regulation annotations [7], and the pattern in this figure shows the effect of a protein on a disease.

## Figures and Tables

**Table 1.** The statistics of LitCovid-AGAC

Name	LitCovid-AGAC
Text type	Title, abstract
Annotation count	AGAC – Var (444), MPA (1,162), CPA (298), NegReg (1,128), PosReg (402), Reg (1,169)
	LitCovid – Mutation (435), Species (152,939), Gene (23,795), Disease (285,135)
	OGER – GO (57,467), CHEBI (111,981)
